# GNSS Signal Tracking Performance Improvement for Highly Dynamic Receivers by Gyroscopic Mounting Crystal Oscillator

**DOI:** 10.3390/s150921673

**Published:** 2015-08-31

**Authors:** Maryam Abedi, Tian Jin, Kewen Sun

**Affiliations:** 1 School of Electronics and Information Engineering, Beihang University, 37 Xueyuan Road Haidian District, Beijing 100191, China; E-Mail: jintian@buaa.edu.cn; 2 School of Computer and Information, Hefei University of Technology, Tunxi Road 193, Hefei 230009, China; E-Mail: kewen.sun@hfut.edu.cn

**Keywords:** highly dynamic GNSS receiver, FLL, gyroscopic mounting, random vibration, sinusoidal vibrations, acoustic

## Abstract

In this paper, the efficiency of the gyroscopic mounting method is studied for a highly dynamic GNSS receiver’s reference oscillator for reducing signal loss. Analyses are performed separately in two phases, atmospheric and upper atmospheric flights. Results show that the proposed mounting reduces signal loss, especially in parts of the trajectory where its probability is the highest. This reduction effect appears especially for crystal oscillators with a low elevation angle g-sensitivity vector. The gyroscopic mounting influences frequency deviation or jitter caused by dynamic loads on replica carrier and affects the frequency locked loop (FLL) as the dominant tracking loop in highly dynamic GNSS receivers. In terms of steady-state load, the proposed mounting mostly reduces the frequency deviation below the one-sigma threshold of FLL (1*σ*_FLL_). The mounting method can also reduce the frequency jitter caused by sinusoidal vibrations and reduces the probability of signal loss in parts of the trajectory where the other error sources accompany this vibration load. In the case of random vibration, which is the main disturbance source of FLL, gyroscopic mounting is even able to suppress the disturbances greater than the three-sigma threshold of FLL (3*σ*_FLL_). In this way, signal tracking performance can be improved by the gyroscopic mounting method for highly dynamic GNSS receivers.

## 1. Introduction

Nowadays, Global Navigation Satellite System (GNSS) receivers are widely used in civil and military highly dynamic vehicles, which need to be accurately and precisely tracked. In general, the dominant signal tracking error sources can be divided into two groups. The first comes from the input signal, which is polluted in the link between the satellite and receiver by thermal noise and the dynamic state of the host vehicle (*i.e.*, velocity, acceleration and jerk), and the second comes from the replica carrier polluted by oscillator inherent error (*i.e.*, Allan deviation), thermal noise and dynamic loads generated by the dynamic state of the host vehicle and vibration loads. The signal will be lost when such errors exceed a certain boundary. Therefore, measurement errors and tracking thresholds are closely related to each other, and both code and carrier tracking loops are nonlinear near the threshold regions [1]. In highly dynamic conditions, dynamic loads degrade both the input signal and the replica carrier. These loads appear as steady-state acceleration, sinusoidal vibration, mechanical and acoustical random vibrations. The effects of these loads on the code tracking loop are negligible; however, these loads are modulated on the replica carrier and cause clock bias. Steady-state load exists all over the trajectory and causes a large phase deviation. When the phase lock is lost, the phase lock detector can distinguish it and transit back to FLL [1]. Thus, the dominant tracking loop in highly dynamic GNSS receivers is FLL. A traditional GPS receiver usually solves the high dynamic problem in baseband signal processing, where the FLL uses a high bandwidth margin for dynamics to avoid signal loss. In addition, the INS-aided method can reduce some of the dynamics applied to the receiver, but it cannot reduce its affinity toward oscillators. Therefore, it is necessary to propose novel methods to reduce the FLL tracking loop threshold.

As described in [2], in highly dynamic conditions, the probability of observing instability in oscillator output is reduced by using the gyroscopic mounting method. In this paper, this mounting is used as a novel error reduction method in the tracking loop of GNSS receivers. In order to prove its positive effects on the signal tracking process, a high dynamic receiver and its corresponding error sources are well defined in Section 2. In Section 3, all dynamic loads and their sources are described in detail. In Section 4, the structure and operation of the aforementioned mounting method is described. In Section 5, the probability of the signal loss is analyzed for atmospheric and upper atmospheric flights. Then, in Section 6, we are going to prove the gyroscopic mounting efficiency for preventing signal loss. To achieve this purpose, the effect of the aforementioned mounting on disturbances resulting from each dynamic load is analyzed separately from Section 6.1, Section 6.2 and Section 6.3. Particularly, we focus on mechanical and acoustical random vibration as the main disturbance sources for FLL from Section 6.3.1, Section 6.3.2 and Section 6.3.3, and we analyze them in the time and frequency domain. In Section 7, the conclusion of the paper is provided.

## 2. Highly Dynamic GNSS Receivers and Error Sources

**Figure 1:**
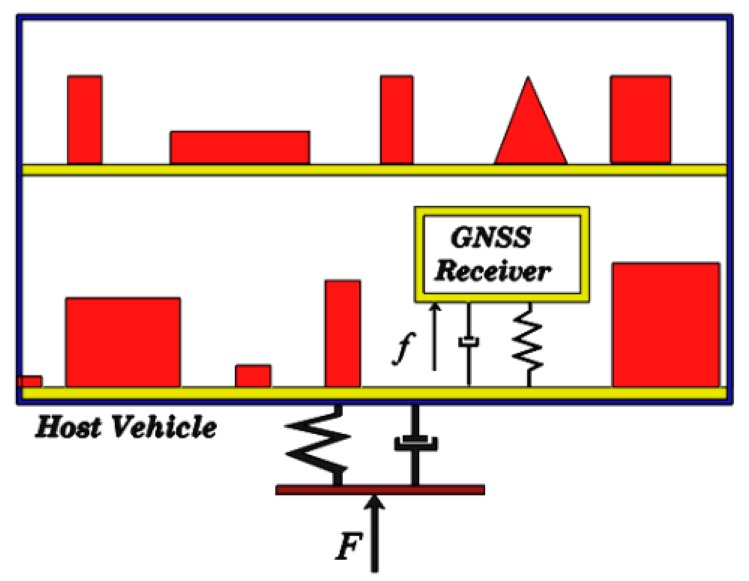
GNSS receiver installed in a highly dynamic host vehicle.

A GNSS receiver installed on any host vehicle moving with acceleration varying with time (*i.e.*, jerk) or space (*i.e.*, circular trajectory) is assigned as a highly dynamic receiver. In this case, a portion of the external loads applied to the host vehicle is denoted by F in Figure 1, and a portion applying to the GNSS receiver installed inside the host vehicle is denoted by f. This portion especially depends on the receiver position inside the host vehicle.

**Figure 2:**
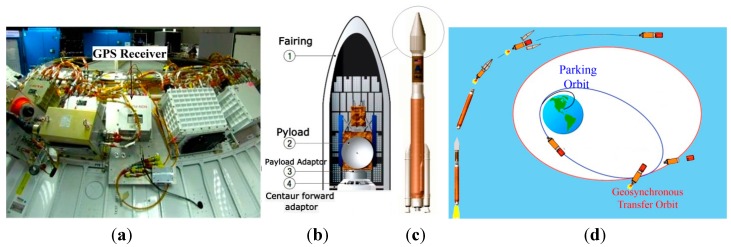
Launch vehicle Atlas V. (**a**) Installation position of GPS receiver on the centaur forward adaptor; adapted from [4]; (**b**) position of the centaur forward adaptor inside the fairing; adapted from [5]; (**c**) position of the fairing in the launch vehicle; adapted from [5]; (**d**) trajectory; adapted from [5].

Generally, host vehicle trajectory includes either atmospheric or upper atmospheric flight. Therefore, in this paper, the launch vehicle experiencing a harsh environment during both atmospheric and upper atmospheric phases is selected as the highly dynamic host vehicle [3]. In this case, as shown in Figure 2, a GPS receiver is installed inside the fairing, on the centaur forward adaptor, where the other electronic equipment is installed [4,5,6].

The testing of the GPS receiver in a real high dynamic launch vehicle is almost impossible. Thus, a real launch vehicle condition is adopted to simulate the high dynamic working environments. In this paper, numerical analyses have been performed on the GPS receiver installed on the Arian launch vehicle, where the GPS L_1_ signal with a carrier frequency of 154 × *ƒ*_0_ (*ƒ*_0_ = 10.23 MHz) is adopted, and the GPS receiver is equipped with an oven control crystal oscillator (OCXO) with a g-sensitivity of *Γ* = 10^−9^/g and Q-factor = 10^11^.

In highly dynamic conditions, the errors affecting signal tracking loops are according to Equations (1)–(4). In these equations, the safety margin (SM) denotes the 3*σ* threshold of FLL and PLL (Phase Locked Loop).

*SM_PLL_* = 3*σ_PLL_* ≤ Phase pullin range of the PLL discriminator/4 = 180°/4 →

(1) $$SM_{PLL}=3\sigma_{PLL}\leq 45{}^{\circ}\;\&\;\frac{SM_{PLL}}{3}=\sigma_{PLL}\leq 15{}^{\circ}$$

(2) $$SM_{PLL}=3\sigma_{PLL}=\theta_{e}+3\sigma_{j}=\theta_{ei}+\theta_{eo}+3\sqrt{{\sigma_{tiPLL}}^{2}+{\sigma_{toPLL}}^{2}+{\sigma_{vPLL}}^{2}+\theta_{A}^{2}}$$

*SM_FLL_* = 3*σ_PLL_* ≤ Frequency pullin range of the FLL discriminator/4 = 1/4 T (T = 20 ms) →

(3) $$SM_{FLL}=3\sigma_{FLL}\leq 12.5\;\left(\text{Hz}\right)\;\&\;\frac{SM_{FLL}}{3}=1\sigma_{FLL}\leq 4.17\;\left(\text{Hz}\right)$$

(4) $$SM_{FLL}=3\sigma_{FLL}=f_{e}+3\sigma_{j}=\;f_{ei}+\;f_{eo}+3\sqrt{{\sigma_{tiFLL}}^{2}+{\sigma_{toFLL}}^{2}+{\sigma_{vFLL}}^{2}+f_{A}^{2}}$$

Incoming signal errors: *θ_ei_* = dynamic stress phase deviation; *ƒ_ei_* = dynamic stress frequency deviation; *σ_tiPLL_* = thermal noise phase jitter; *σ_tiFLL_* = thermal noise frequency jitter.

Replica carrier errors: *θ_eo_* = dynamic stress phase deviation; *ƒ_eo_* = dynamic stress frequency deviation; *σ_vPLL_* = vibration-induced phase jitter; *σ_vFLL_* = vibration-induced frequency jitter; *θ_A_* = Allan deviation-induced phase jitter; *ƒ_A_* = Allan deviation-induced frequency jitter [7]; *σ_toPLL_* = thermal noise phase jitter [7]; *σ_toFLL_* = thermal noise frequency jitter.

## 3. Dynamic Loads Sources and Impacts on the Replica Carrier

In highly dynamic vehicles, dynamic loads can be usually classified into steady-state acceleration, sinusoidal vibrations, mechanical and acoustical random vibration. These loads sources could be considered as the following [5,8]:

- Trust is the propulsion load, which is generated by engines minus the drag forces in the trajectory, which appears as steady-state acceleration.
- Rotating devices, such as engines, generate sinusoidal vibrations existing during powered flight, and this is in the maximum state during atmospheric flight and at the points of the start and shut down of the engines.
- Sound pressure load is generated by plumes, boundary layer turbulence, air separation and shock waves. This load appears as acoustical random vibration.
- Separation of the boosters, engines and fairings causes a shock load on the launch vehicle, appearing as mechanical random vibration [5]*.*

**Figure 3:**
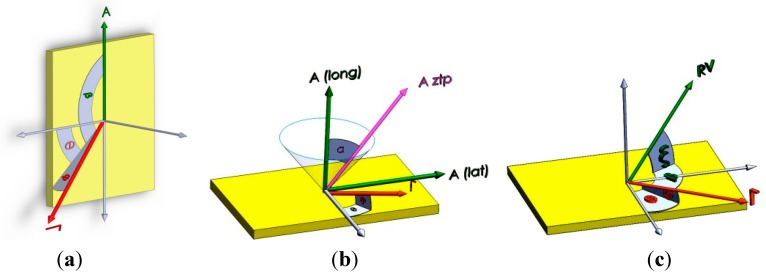
Clock bias sources for reference oscillators of highly dynamic receivers. (**a**) Steady-state acceleration, deviation source; (**b**) sinusoidal vibration, jitter source; (**c**) random vibration, jitter source.

Dynamic loads are the clock bias sources in the form of frequency and phase errors, as shown in Figure 3. The usual expressions for these disturbances are:

Frequency error:

(5) $$\text{Δ}f=154\times f_{0}\vec{\text{Γ}}.\vec{A}=154\times f_{0}\text{Γ}A\;cos\alpha \;cos\beta$$

Phase error:

(6) $$\text{Δ}\varphi =\;2\pi \;\int \text{Δ}fdt=2\pi \times 154\times f_{0}\text{Γ}.\;\int Adt=2\pi \times 154\times f_{0}\text{Γ }cos\alpha \;cos\beta \int Adt\;$$

where, according to Figure 3, 0 < |*β*| < π, 0 < |*α*| < π; *A* = dynamic load; *α* = the angle between load *A* and g-sensitivity vector *Γ*; *β* = angle between pages through the load *A* and g-sensitivity vector *Γ*.

## 4. Gyroscopic Mounting on the GPS Board

**Figure 4:**
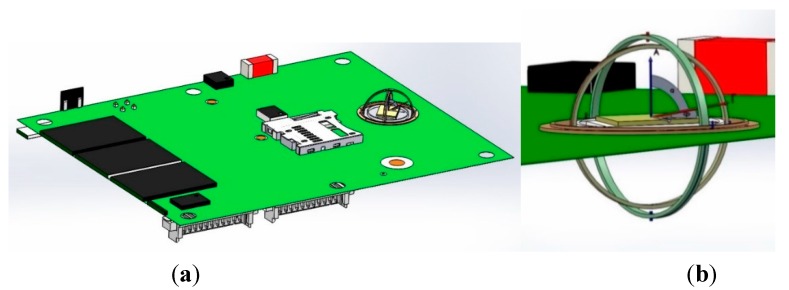
GPS receiver electronic board equipped with the gyroscopic mounting instrument. (**a**) Overall view; (**b**) detailed view.

**Figure 5:**
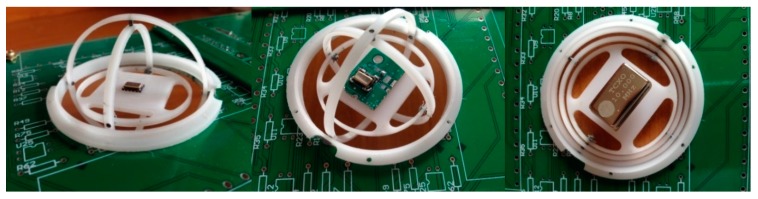
GPS receiver crystal oscillators on the engineering model of the gyroscopic mounting.

As described in detail in [2], this mounting is an instrument similar to a gyroscope. It can be used to install the GPS receiver’s reference oscillator on a PCB, as shown by the modeling in Figure 4 and by the engineering model (EM) in Figure 5, respectively. This mounting is proposed as a novel error reduction method in the tracking loop for GNSS receivers in order to reduce the impacts of dynamic loads on the replica carrier. When the combination of dynamic loads is applied to the oscillator on some parts of the host vehicle trajectory, the gyro rotates in such a way that the resultant load is perpendicular to the crystal surface.

## 5. Trajectory and Signal Loss

**Figure 6:**
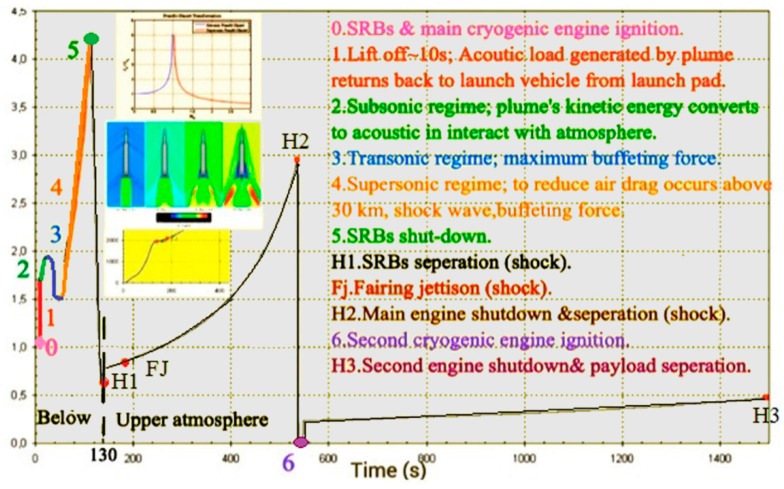
Sequence of events on the trajectory (SBR = Solid Rocket Booster).

**Figure 7:**
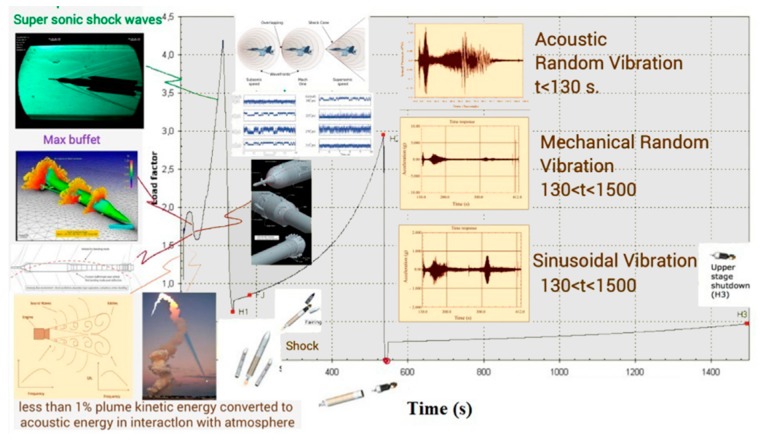
External loads applied to the Ariane 5 launch vehicle.

**Figure 8:**
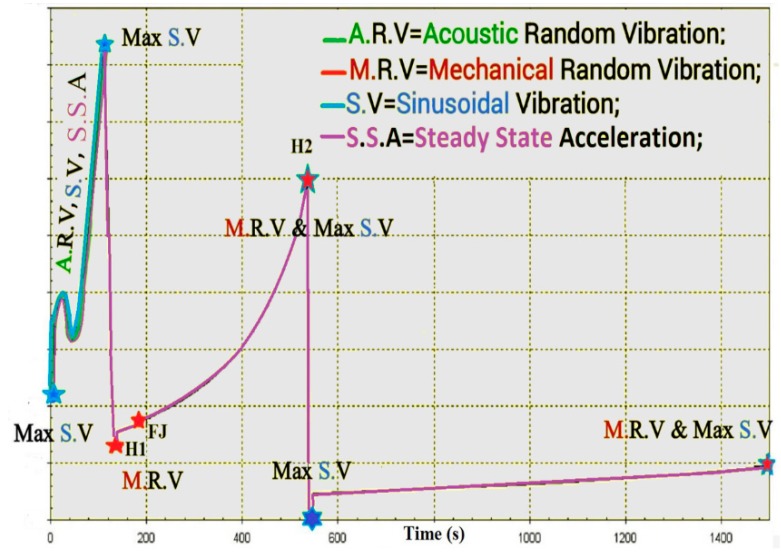
Dynamic loads applied to the Aian5 payload over the trajectory.

In highly dynamic GNSS receivers, the probability of signal loss is different in each step of flight. For the ease of understanding, dynamic loads and their sources are shown on the trajectory of the Arian 5 in Figure 6, Figure 7 and Figure 8 [6,8,9,10,11,12,13,14,15,16,17,18]. These data are applied to estimate where the probability of tracking loss is the highest. Then, we can simply find the main reasons for the signal loss and the disturbance sources to either damp these sources or to reduce their impacts on the replica carrier.

Atmospheric flight is defined as the flight path within the atmosphere (*H* < 100 km). This happens for the Arian 5 during *t* < 130 s. In this phase, steady-state load, big sinusoidal vibration and acoustical random vibrations are the dominant loads.

Upper atmospheric flight is defined as the flight path outside the atmosphere (*H* > 100 km). This happens for the Ariane 5 during 130 < *t* < 1500. In this phase, steady-state load, sinusoidal vibration and mechanical random vibrations exist over the trajectory. However, vibration loads are in the maximum state during short periods, as shown in Figure 6 in detail.

**Figure 9:**
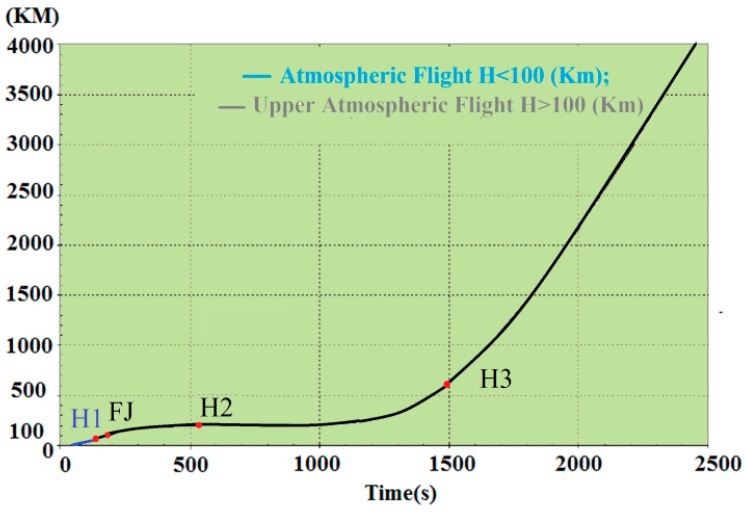
Arian 5 sequences of events during atmospheric and upper atmospheric flight, adapted from [8].

According to Figure 8, it is easy to understand that maximum signal loss happens during atmospheric flight and in some intervals in upper atmospheric flight when maximum loads are combined together. These points are illustrated by the star in Figure 8.

According to Figure 6, Figure 7 and Figure 8, the trajectory can be divided into two parts, atmospheric and upper atmospheric flights, as illustrated in Figure 9.

## 6. Dynamic Loads Impact the Replica Carrier and the Proof of Gyroscopic Mounting Efficiency

### 6.1. Steady-State Acceleration (g)

**Figure 10:**
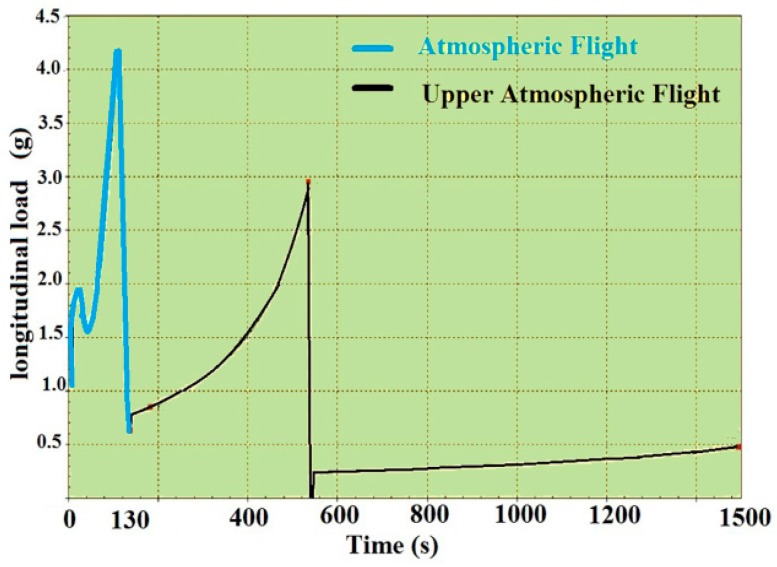
Longitudinal steady-state acceleration of the Ariane 5 launch vehicle, adapted from [8].

The acceleration of the host vehicle on the trajectory can be divided into either longitudinal (trust) or lateral directions (maneuver) [8]. As the density of air and the mass of the vehicle change on each step of flight, this load is a time-variant acceleration (jerk). For the Ariane 5 launch vehicle, the longitudinal load is illustrated in Figure 10. Since this load presents all over the trajectory, it affects both the atmospheric and upper atmospheric signal loss.

**Figure 11:**
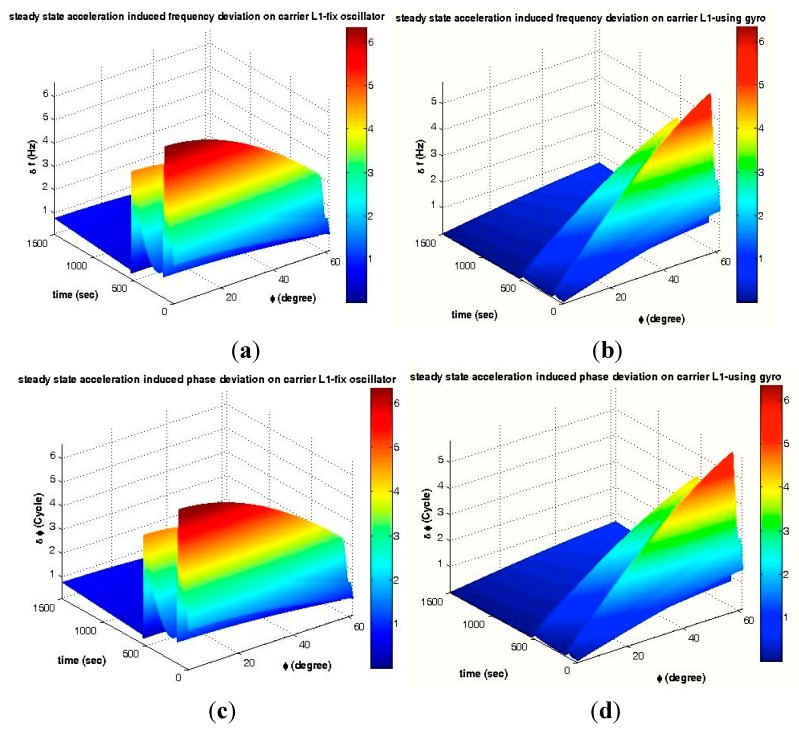
Maximum disturbances caused by steady-state acceleration. (**a**) Frequency deviation of a fixed oscillator; (**b**) Frequency deviation of the gyroscopic mounting; (**c**) Phase deviation of a fixed oscillator; (**d**) Phase deviation of the gyroscopic mounting.

Steady-state load-induced disturbances on the replica carrier, before and after using the gyroscopic mounting, are expressed as below. Quiet often, the restrictions on the installation of the receiver on the vehicle affect these disturbances. The maximum state is shown in Figure 3a.

(7) $$\text{Δ}f_{fixed\text{-}xo}\left(\tau =1\text{ s}\right)=154\times f_{0}\text{Γ}A\;cos\alpha \;cos\beta ;\text{ Δ}\varphi_{fixed\text{-}xo}\left(Bn=1\text{ Hz}\right)=2\pi \text{ Δ}f_{fixed-xo}$$

(8) $$\text{Δ}f_{gyro}\left(\tau =1\text{ s}\right)=154\times f_{0}\text{Γ}A\;sin\varphi ;\text{ Δ}\varphi_{gyro}\left(Bn=1\text{ Hz}\right)=2\pi \text{ Δ}f_{gyro}$$

where 0 < |*β*| < π and 0 < |*α*| < π, as shown in Figure 3a; *A* is defined according to Figure 10. The critical state and max gyroscopic mounting effect appear when *β* = *k*π (*k* = 0, 1). Based on Equations (7) and (8), the frequency and phase deviation with and without the gyroscopic mounting are shown in Figure 11.

As shown in Figure 11c, this load causes great phase deviation in comparison to the PLL thresholds defined in Equation (1). Therefore, the phase lock is lost, and the sensitive phase lock detector distinguishes it and transits back to FLL [1]. That is why the dominant tracking loop in highly dynamic receivers is FLL.

**Table 1:** Gyroscopic mounting effect on steady-state load-induced disturbances on FLL for the most probable crystals.

Crystal Specifications (Figure 3a)	*β* = 0, |*φ*| = 2		Gyro Effect	*β* = 0, |*φ*| = 38°		Gyro Effect
Oscillator status	Fixed	on Mounting		Fixed	on Mounting	
**Atmospheric Flight** Δƒ**(Hz)**	$6.61>\frac{SM_{FLL}}{3}$	$0.23<<<\frac{SM_{FLL}}{3}$	**(96.52%)**	$5.21>\frac{SM_{FLL}}{3}$	$4.07<\frac{SM_{FLL}}{3}$	**(21.88%)**
**Upper-Atmospheric Flight** Δƒ**(Hz)**	$4.72>\frac{SM_{FLL}}{3}$	$0.16<<<\frac{SM_{FLL}}{3}$	**(96.612%)**	$3.72<\frac{SM_{FLL}}{3}$	$2.91<<\frac{SM_{FLL}}{3}$	**(21.77%)**

As seen in Figure 11a, either during the atmospheric or upper atmospheric mission, the maximum frequency deviation would be greater than the one-sigma threshold of FLL (4.17 Hz). According to Figure 7, the combination of different dynamic loads applied to the oscillator and disturbances caused by other dynamic loads are added to this disturbance. As shown in Figure 11b, the gyroscopic mounting presents good performance and reduces the maximum disturbances of the atmospheric and upper atmospheric flights, especially for crystals with a low g-sensitivity vector elevation angle [2]. In a simple sense, in this way, the gyroscopic mounting prevents the signal loss caused by the steady-state load in most cases. The values of the disturbances are shown in Table 1 for the most probable crystals, *i.e.*, |*φ*| < 38° = 2*σ* (84%) [2], during the atmospheric and upper atmospheric flights.

### 6.2. Sinusoidal Vibrations (2–100 Hz)

**Figure 12:**
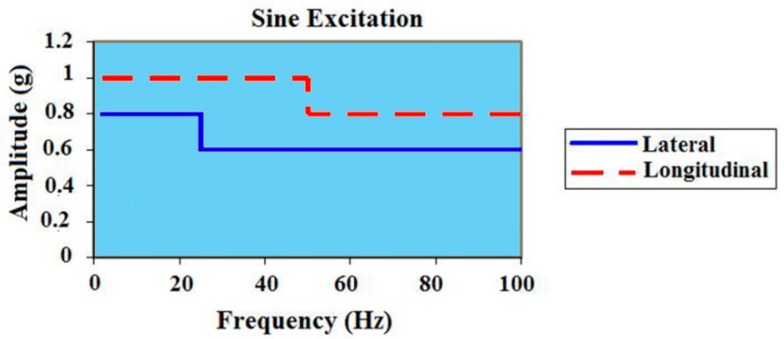
Arian 5 sinusoidal vibration, adapted from [8].

This is a sweep of all of the sinusoidal excitations of a frequency of 2 Hz–200 Hz with different amplitudes in the longitudinal and lateral directions [8,9,10] for the Ariane 5, as shown in Figure 12. The maximum sinusoidal vibration exists during atmospheric flight and at the points of the engine’s start and shutdown. Since every rotating device, such as the engine, causes sinusoidal vibration, this load exists all over the trajectory with different amplitudes in each phase [8].

Sinusoidal vibration-induced disturbances on the replica carrier, before and after using the gyroscopic mounting, are expressed as:

(9) $$\text{Δ}f_{fixed-xo}(\tau =1/f_{v})\leq 154\times f_{0}\text{Γ}A_{ztp}\sin(\alpha +\varphi )cos\beta ;\text{ Δ}\varphi_{fixed-xo}(Bn=f_{v}\text{ Hz})=\text{Δ}f/f_{v}$$

(10) $$\text{Δ}f_{gyro}(\tau =1/f_{v})\leq 154\times f_{0}\text{Γ}A_{ztp}\sin\varphi ;\text{ Δ}\varphi_{gyro}(Bn=f_{v}\text{ Hz})=\text{Δ}f_{gyro}/f_{v}$$

where 0 < |*β*| < π; *A*_ztp_ and *α* can be calculated from the lateral and longitudinal load, respectively, for each frequency band. The critical state and the max gyro effect appear when *β* = *k*π (*k* = 0, 1…).

**Figure 13:**
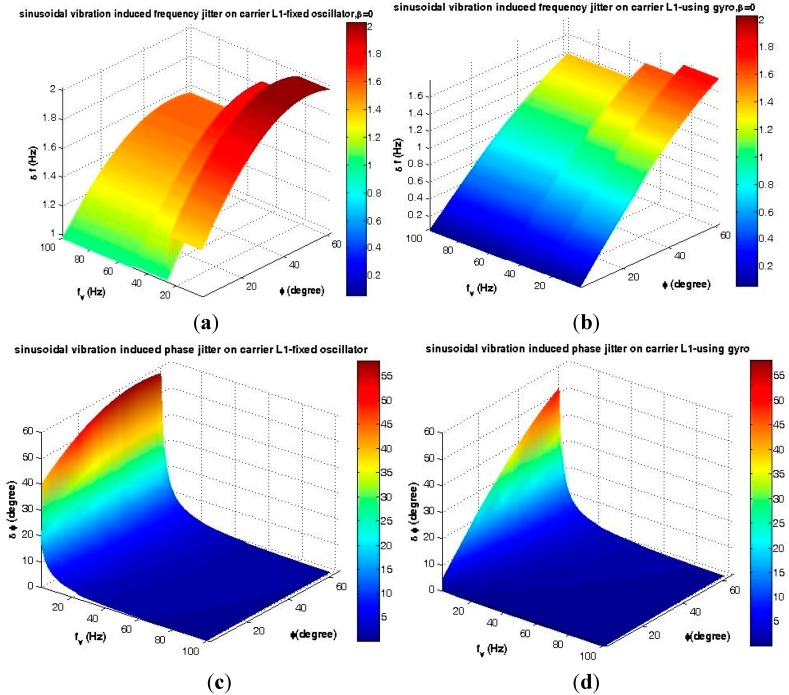
Maximum disturbances caused by sinusoidal vibration. (**a**) Frequency jitter of a fixed oscillator; (**b**) Frequency jitter of the gyroscopic mounting; (**c**) Phase jitter of a fixed oscillator; (**d**) Phase jitter of the gyroscopic mounting.

Based on Equations (9) and (10), the frequency and phase deviation with and without the gyroscopic mounting are shown in Figure 13.

As seen in Figure 13c, low frequency sinusoidal vibrations induce big phase jitter in comparison to the PLL threshold, *i.e.*, 1*σ*_PLL_ = 15°. However, as described in Section 6.1.1, highly dynamic receivers only benefit FLL as the tracking loop, and fortunately, as shown in Figure 13a, the sinusoidal vibration-induced frequency jitter is below the 1*σ*_FLL_ threshold, *i.e.*, 4.17 Hz. Nevertheless, according to Figure 8, in some parts of the trajectory, this small jitter is combined with disturbances caused by the other dynamic loads and also with the other disturbance sources described in Equations (1)–(4) and, consequently, causes signal loss. By using the proposed mounting method, this frequency jitter is significantly reduced, as can be seen in Figure 13b, and signal loss is prevented when the other error sources are accompanied with sinusoidal vibrations. According to Figure 13a–d, the strongest point of this mounting is that its best effects appear for the most probable crystals, *i.e.*, the g-sensitivity elevation angle |*φ*| ≤ 38°, as described in detail in [2] and Table 2.

**Table 2:** Gyroscopic mounting effect on sinusoidal vibration-induced disturbances on FLL at critical points.

Crystal Specifications (Figure 3)	*β* = 0, |*φ*| = 2		*β* = 0, |*φ*| = 38°		*β* = 0, |*φ*| = 51°	
Oscillator Status	Fixed	on Mounting	Fixed	on Mounting	Fixed	on Mounting
**Δƒ (Hz)**	$1.32<\frac{SM_{FLL}}{3}$	$0.070<<\frac{SM_{FLL}}{3}$	$1.96<\frac{SM_{FLL}}{3}$	$1.24<<\frac{SM_{FLL}}{3}$	$2<\frac{SM_{FLL}}{3}$	$1.57<<\frac{SM_{FLL}}{3}$
**Gyro effect**	**94.70%**		**36.73%**		**21.50%**	

### 6.3. Random Vibration

Generally, random vibration is a bandlimited noise following the Gaussian distribution and combined with all of the excitations of 20–2000 Hz for mechanical ones and 20–10,000 Hz for acoustical ones [5,8,9,10,19,20,21,22,23].

#### 6.3.1. Affinity of Random Vibration on the Replica Carrier

Mechanical random vibration arise from shock load generated by the separation of boosters, engines and fairings in the case of the launch vehicle.

Acoustical random vibration arises from acoustic pressure load generated by different sources depicted and explained in Figure 6 and Figure 7 during atmospheric flight, *i.e.*, *t* < 130 s for the Ariane launch vehicle. According to these figures, during the transonic and lift-off phases (*i.e.*, *t* < 10 s and 20 s < *t* < 60 s), this load is in the maximum state.

**Figure 14:**
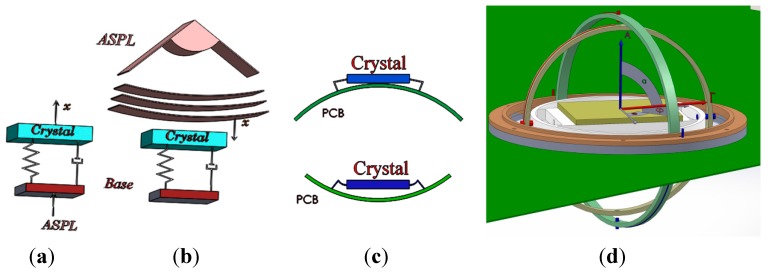
Acoustic load applied on the crystal (**a**) as the mechanical random vibration from the base and (**b**) as the acoustical random vibration directly on the surface; (**c**) the effect of flexible connectors on transferring random vibration from the base; (**d**) The isolator (LPF) between main ring of the gyroscopic mounting and the PCB.

Acoustical random vibration affects the crystal oscillator output in two ways, as shown in Figure 14a,b [5,9,24]:

- Acoustic load can be applied directly on any surface and generates acoustical random vibration. In some electronic devices, like the crystal oscillator, this random vibration converts to Δ*v*, the so-called microphonic effect. This effect causes disturbances in the oscillator output as frequency and phase jitter.
- Acoustic load is converted to mechanical random vibration on the plates, such as PCBs, and transfers to the equipment installed on them, such as the crystal oscillator from connectors. This part of the random vibration is very small in comparison to the direct one [5]. In a simple sense, for a thinner and broader PCB, stronger random vibration is generated; also for a more flexible connection between the oscillator and PCB, less random vibration transfers to the crystal, as can be seen in Figure 14c [24]. In the case of the gyroscopic mounting, this portion of the acoustic random vibration is decreased greatly. However, this load can be reduced more by installing a one-layer isolator beneath the main ring of the mounting, where it is installed on the PCB, as shown in Figure 14d. The isolator with considerable low stiffness acts as the low pass filter with a low stop band frequency [25].

#### 6.3.2. Analysis of the Random Vibration in the Time Domain

##### Mechanical Random Vibration

**Figure 15:**
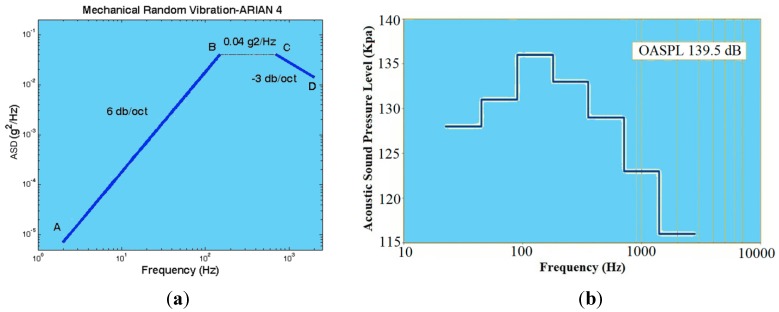
Spectrum for inside the fairing of the Ariane launch vehicle. (**a**) Acceleration spectral density (ASD; g^2^/Hz) for mechanical random vibration, adapted from [20]; (**b**) noise spectrum (dB–Hz) for acoustical random vibration, adapted from [8].

Mechanical random vibration is defined by ASD (acceleration spectral density) in the frequency domain, as shown in Figure 15a. According to Parseval’s law, 1*σ* of this acceleration in the time domain is equal to *g*_rms_:

(11) $$g_{rms}=\sqrt{ASD\times f}$$

(12) $$\text{From Point A to B}:20<f<150,ASD=1.7778\times 10^{-6}f^{2}$$

(13) From Point B to C:150≤f<700,ASD=0.04

(14) $$\text{From Point C to D}:700\leq f<2000,PSD=\frac{28}{f}$$

(15) $$g_{rms}(1\sigma )=7.3\;(\text{g})$$

##### Acoustical Random Vibration

Acoustical random vibration is defined by the spectrum (dB–Hz) in the frequency domain, as shown in Figure 15b*.* A similar picture holds for 1*σ* of this random vibration load. According to Parseval’s law, the amplitude of this load in the time domain is sound pressure (kPa); and 1*σ* of this vibration is equal to *P*_rms_, which can be calculated as follows:

(16) $$\begin{array}{l} \text{Overall Acoustic Sound Pressure Load }(\text{OASPL})\;=20\;log\left(P_{rms}/P_{ref}\right)\\ \text{OASPL}=139.5\;\left(\text{dB}\right);\;f=20\sim 2828\;\left(\text{Hz}\right);\;P_{ref}=20\;\left(\text{μPa}\right) \end{array}$$

(17) $$P_{rms}(1\sigma )=0.189\;\left(\text{kPa}\right)$$

**Figure 16:**
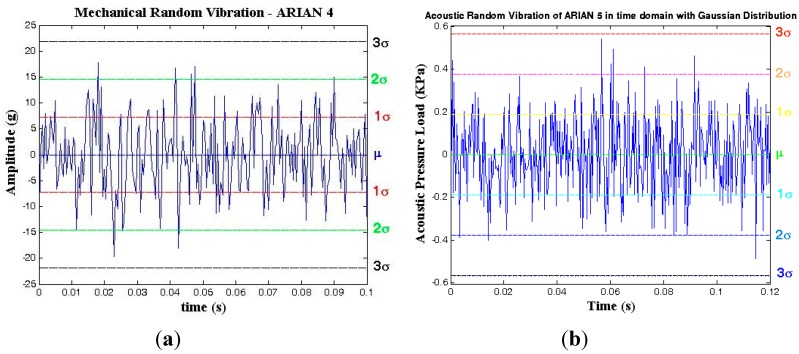
Time domain representation of the random vibration with the Gaussian distribution for the Arian launch vehicle. (**a**) Mechanical random vibration (20–2000 Hz); (**b**) acoustical random vibration (20–2828 Hz).

According to Equations (11)–(17), the time domain representation of random vibration for Ariane launch vehicle can be seen in Figure 16.

**Figure 17:**
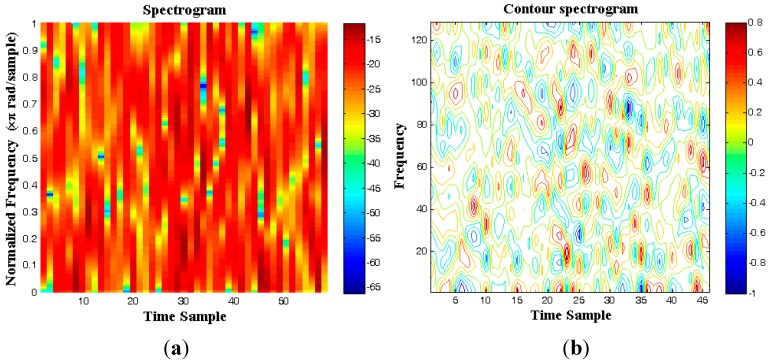
Spectrogram of the acoustical random vibration inside the fairing of the Ariane launch vehicle; hamming window: 16. (**a**) Spectrogram; (**b**) contour of the spectrogram.

The spectrogram of acoustical random vibration is shown in Figure 17a,b for 0.12 s by choosing a hamming window of 16. By comparison of Figure 16b and Figure 17a,b, the normal distribution of the random vibration is evident. A similar picture holds for the mechanical random vibration.

#### 6.3.3. Analysis of the Gyroscopic Mounting Effects on the Random Vibration-Induced Disturbances in the Replica Carrier L_1_ in the Time Domain

##### Mechanical Random Vibration

(18) $$\begin{array}{l} \text{Δ}f_{fixed-xo}\left(\tau =1/f_{RV}\right)=154\times f_{0}\text{Γ}A_{mrv}\;cos\left(\xi -\varphi \right)\;cos\beta \;\\ \text{Δ}\varphi_{fixed-xo}\left(B_{n}=f_{RV}\right)=154\times 2\pi \;\times f_{0}A_{mrv}\text{Γ}cos\left(\xi \;-\varphi \right)\;cos\beta \;\sum cos\;\left(2\pi f_{vi}.t\right)/2\pi f_{vi}=\text{Δ}ƒ/f_{RV} \end{array}$$

(19) $$\text{Δ}f_{gyro}\left(\tau =1/f_{RV}\right)=154\times f_{0}\text{Γ}A_{mrv}\;sin\varphi ;\text{ Δ}\varphi_{gyro}\left(B_{n}=f_{RV}\right)=\text{Δ}f_{gyro}/f_{RV}$$

where 0 < |*φ*| < π/2, 0 < |*β*| < π and 0 < |*ξ*| < π, as shown in Figure 3c; the amplitude of the mechanical random vibration, *A_mrv_* is between −3*σ* and 3*σ*, as shown in Figure 16a; the critical state and maximum effects of the gyroscopic mounting appear when *β* = *k*π and *ξ − φ* = *k*π.

##### Acoustical Random Vibration

The acceleration can be calculated from the acoustic pressure load in consideration of the mass, area and acoustic absorption coefficient [5] of the crystal blank.

(20) $$A_{a}\left(g\right)=P_{arv}\times \alpha \times k/9.8$$

(21) $$\begin{array}{l} \text{Δ}f_{fixed-xo}\left(\tau =1/f_{RV}\right)=154\times f_{0}\text{Γ}A_{a}\;cos\left(\xi -\varphi \right)\;cos\beta \;\\ \text{Δ}\varphi_{fixed-xo}\left(B_{n}=f_{v}\right)=154\times 2\pi \times f_{0}A_{a}\text{ Γ}cos\left(\xi \;-\varphi \right)\;cos\beta \;\sum cos\;\left(2\pi f_{vi}.t\right)/2\pi f_{vi}=\text{Δ}f/f_{RV} \end{array}$$

(22) $$\text{Δ}f_{gyro}\left(\tau =1/f_{RV}\right)=154\times f_{0}\text{Γ}Asin\varphi ;\text{ Δ}\varphi_{gyro}\left(B_{n}=f_{RV}\right)=\text{Δ}f_{gyro}/f_{RV}$$

where *A*_a_ is the acceleration applied by the acoustic load; *P_arv_* is the amplitude of the acoustical random vibration, and it is between −3*σ* and 3*σ*, as shown in Figure 16b; *α* = the sound absorption coefficient of the crystal; *k* = the surface area/crystal mass; 0 < |*φ*| < π/2, 0 < |*β*| < π and 0 < |*ξ*| < π, as shown in Figure 3c; the critical state and maximum effects of the gyroscopic mounting appear when *β* = *k*π and *ξ – φ* = *k*π.

#### 6.3.4. Results and Analysis

##### Mechanical Random Vibration

**Figure 18:**
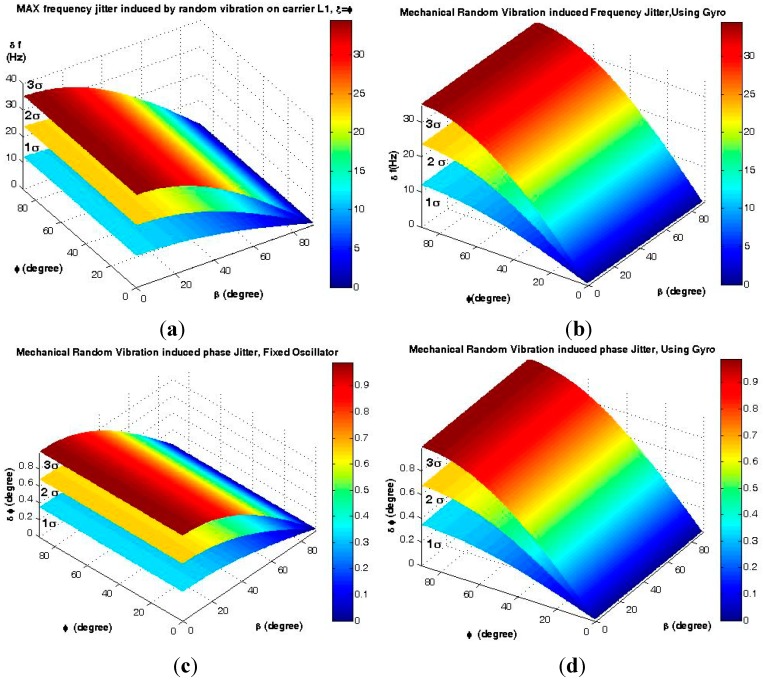
Time domain analysis of the disturbances induced by mechanical random vibration for *ξ* = *φ*, 0 < *β* < 90° and *ƒ_RV_* = 2000 Hz. (**a**) Frequency jitter of a fixed oscillator; (**b**) Frequency jitter of the gyro mounting; (**c**) Phase jitter of a fixed oscillator; (**d**) Phase jitter of the gyro mounting.

**Figure 19:**
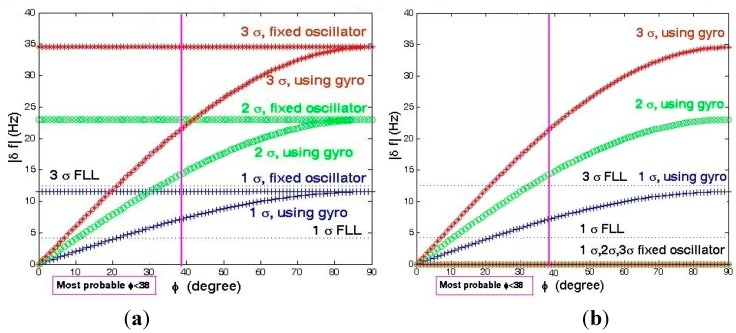
Analysis of FLL for receivers equipped with the gyroscopic mounting in different states. (**a**) Maximum effect state *ξ* = *φ*, *β* = 0; (**b**) Maximum drawback state, *ξ – φ* = 90° or *β* = 90°.

The maximum effects and drawbacks of the proposed mounting on the mechanical random vibration-induced frequency jitter during the upper atmospheric signal tracking process are shown in Figure 18 and Figure 19 and Table 3.

**Table 3:** Gyroscopic mounting effect on mechanical random vibration-induced disturbances on FLL.

Amplitude of RV	Mechanical Random Vibration–Induced Frequency Jitter (Hz)			
	Max Gyro Effect *ξ* = *φ*, *β* = 0, *φ* = 0 (Figure 3c) Figure 19a		Max Gyro Drawback *ξ − φ* = 90 or *β* = 90°, |*φ*| = 38° (Figure 3c) Figure 19b	
	Fixed Oscillator	Using Gyro	Fixed Oscillator	Using Gyro
**1*σ* (68.3%)**	$11.51\approx SM_{FLL}$	**0**	**0**	$\frac{1}{3}SM_{FLL}<7.09<\frac{2}{3}SM_{FLL}$
**2*σ* (95.6%)**	$23.03>>SM_{FLL}$	**0**	**0**	$14.18\geq SM_{FLL}$
**3*σ* (99.7%)**	$34.54>>>SM_{FLL}$	**0**	**0**	$21.26>>SM_{FLL}$

According to Figure 3c, in the critical state (*β* = 0 and *ξ* = *φ*), *i.e.*, when the load coincides with the g-sensitivity vector, this mounting is able to reduce the frequency jitter caused by vibrations with amplitudes ≤1*σ* (*i.e*., nearly 68% of the polluted trajectory with this load), from values close to the 3*σ*_FLL_ threshold to nearly close to or below the 1*σ*_FLL_ threshold for crystals with a g-sensitivity angle |*φ*| ≤ 38° [2]. In the safe state (*β* = 90° or *ξ – φ* = 90°), *i.e.*, when the load is perpendicular to the g-sensitivity vector, this mounting causes some disturbances to the tracking loop. Nevertheless, this drawback is less than 1*σ*_FLL_ (4.17 Hz) for crystals with a g-sensitivity elevation angle less than 20° (= 1*σ* = 42% of cases [2]), and it is between 1*σ*_FLL_ and 2*σ*_FLL_ (8.34 Hz) for crystals with the g-sensitivity elevation angle less than 38° (= 2*σ* = 84% of cases [2]). Therefore, the gyro does not cause signal loss on nearly 68% of the polluted trajectory by mechanical random vibration. Further, it is worth remembering that these drawbacks only occur when *β* or *ξ – φ* are close to 90°. In a simple sense, the probability of its occurrence is sufficiently low.

##### Acoustical Random Vibration

According to Equations (18)–(20), the necessary parameters to calculate load *A*_a_ are the sound absorption coefficient *α* and *k* = Surface Area/Mass of crystal. Therefore, numerical analysis of the acoustical random vibration depends on the mentioned parameters. Whatever this load is, a kind of random vibration, it can be deduced that the response of the gyroscopic mounting to this load is the same as its response to mechanical random vibration analyzed above.

#### 6.3.5. Analysis of the Random Vibration in the Frequency Domain

##### Mechanical Random Vibration

Since, according to Figure 15a, different ASDs with the same *g*_rms_ generate the same vibration in the time domain, therefore, besides the analysis in the time domain, analysis in the frequency domain is required.

(23) $$RV_{\max}=\sqrt{2}g_{rms}=\sqrt{2ASD\times f}$$

(24) $$\text{From Point A to B: }20<f<150,\;RV_{\max}=0.0019\times f^{1.5}\;(\text{g})$$

(25) $$\text{From Point B to C: }150\leq f<700,\;RV_{\max}=0.2828\times f^{0.5}\;(\text{g})$$

(26) $$\text{From Point C to D: }700\leq f<2000,\;RV_{\max}=7.4833\;(\text{g})$$

##### Acoustical Random Vibration

Since, according to Figure 15b, different acoustic load spectrums with the same *P*_rms_ generate the same vibration in the time domain, besides analyses in the time domain, it is necessary to analyze this in the frequency domain.

(27) $$f_{c}=\sqrt{f_{\min}.f_{\max}};\;(f=20-3125\text{ Hz})$$

(28) $$SPL=20\;log\left(P_{rms}/P_{ref}\right)$$

(29) $$RV_{ztp}=\sqrt{2}\;P_{rms}$$

where *SPL* is the Sound Pressure Level, *P_ref_* = 2 × 10^−5^ Pa; *P_rms_* for each frequency band;

**Table 4:** Acoustic noise spectrum under the fairing of Arian 5.

Octave Center Frequency (Hz)	Frequency Band (Hz)	Sound Pressure Level (dB)	*P_rms_* (Pa)	*RV_ztp_* (Pa)
31.5	20–49.61	128	50.24	71.05
63	49.61–80	131	70.96	100.36
125	80–195.31	136	126.19	178.46
250	195.31–320	133	89.34	126.34
500	320–781.25	129	56.37	79.72
1000	781.25–1280	123	28.25	39.95
2000	1280–3125	116	12.62	17.85

#### 6.3.6. Analysis of the Gyroscopic Mounting Effects on the Random Vibration-Induced Disturbances in the Replica Carrier L_1_ in the Frequency Domain

##### Mechanical Random Vibration

(30) $$\begin{array}{l} \text{Δ}f_{fixed-xo}\left(\tau =1/f_{v}\right)\leq 154\times f_{0}\text{Γ }RV_{Max}cos\left(\xi -\varphi \right)\;cos\beta \\ \text{Δ}\varphi_{fixed-xo}\left(Bn=f_{v}\text{ Hz}\right)=154\times 2\pi \times f_{0}\text{Γ}cos\left(\xi -\varphi \right)\;cos\beta \;RV\int \;sin\left(2\pi f_{v}t\right)dt\leq \text{ Δ}f/f_{v} \end{array}$$

(31) $$\text{Δ}f_{gyro}\left(\tau =1/f_{v}\right)=154\times f_{0}\text{Γ }RV_{MAX}sin\varphi ;\text{ Δ}\varphi_{gyro}\left(B_{n}=f_{v}\text{ Hz}\right)\leq \text{Δ}f_{gyro}/f_{v}$$

where *RV_max_* is calculated according to Equations (22)–(26); 20 < *ƒ*_v_ < 2000 Hz; 0 < |*β*| < π, 0 < |*ξ*| < π and 0 < |*φ*|< π/2, as shown in Figure 3c; the critical state and maximum gyro effect appear when *β* = *k*π and *ξ – φ* = *k*π (*k* = 0, 1).

##### Acoustical Random Vibration

Load A can be calculated from *RV*_ztp_ in consideration of the mass, area and acoustic absorption coefficient [4] of the crystal blank.

(32) $$A\left(g\right)=RV_{ztp}\times \alpha \times k/9.8$$

(33) $$\begin{array}{l} \text{Δ}f_{fixed-xo}\left(\tau =1/f_{v}\right)\leq 154\times f_{0}\text{Γ A }cos\left(\xi -\varphi \right)\;cos\beta \\ \text{Δ}\varphi_{fixed-xo}\left(Bn=f_{v}\text{ Hz}\right)=154\times 2\pi \times f_{0}\text{Γ}cos\left(\xi -\varphi \right)\;cos\beta \;RV\int \;sin\left(2\pi f_{v}t\right)dt\leq \text{ Δ}f/f_{v} \end{array}$$

(34) $$\text{Δ}{ƒ}_{gyro}\left(\tau =1/f_{v}\right)=154\times f_{0}\text{Γ }Asin\varphi ;\text{ Δ}\varphi_{gyro}\left(B_{n}=f_{v}\text{ Hz}\right)\leq \text{Δ}f_{gyro}/f_{v}$$

where *A* is the acceleration applied to the oscillator by the acoustic load; *RV_ztp_* is indicated in Table 4; *α* is the sound absorption coefficient of the crystal; *k* = Surface Area/Mass of crystal; 20 < *ƒ_v_* < 3125 Hz; 0 < |*β*| < π, 0 < |*ξ*| < π and 0< |*φ*| < π/2, as shown in Figure 3c; the critical state and maximum gyro effect appear when *β* = *k*π and *ξ – φ* = *k*π (*k* = 0, 1).

#### 6.3.7. Results and Analysis

##### Mechanical Random Vibration

**Figure 20:**
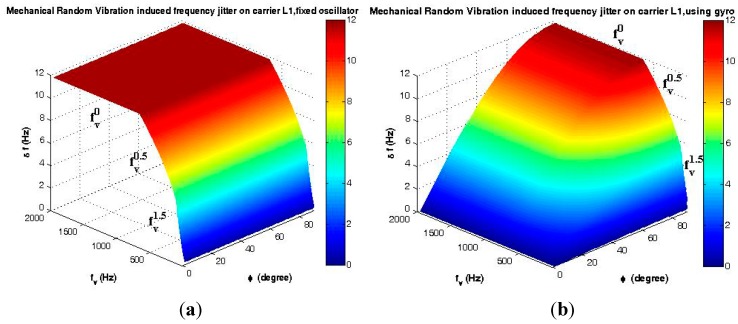

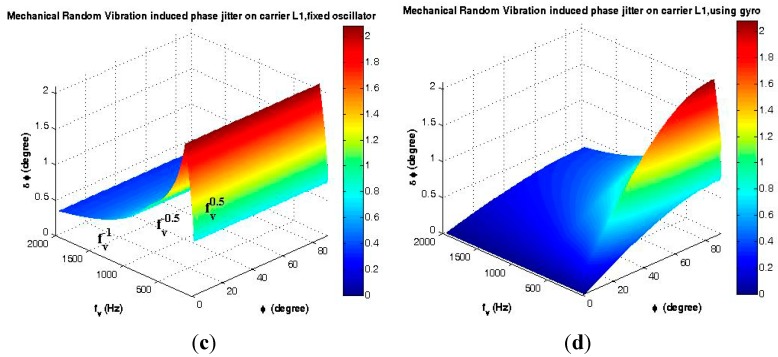
Analysis of the maximum mechanical random vibration in the frequency domain for *β* = 0 and *ξ* = *φ*. (**a**) Frequency jitter of a fixed oscillator; (**b**) Frequency jitter of the gyroscopic mounting; (**c**) Phase jitter of a fixed oscillator; (**d**) Phase jitter of the gyroscopic mounting.

**Figure 21:**
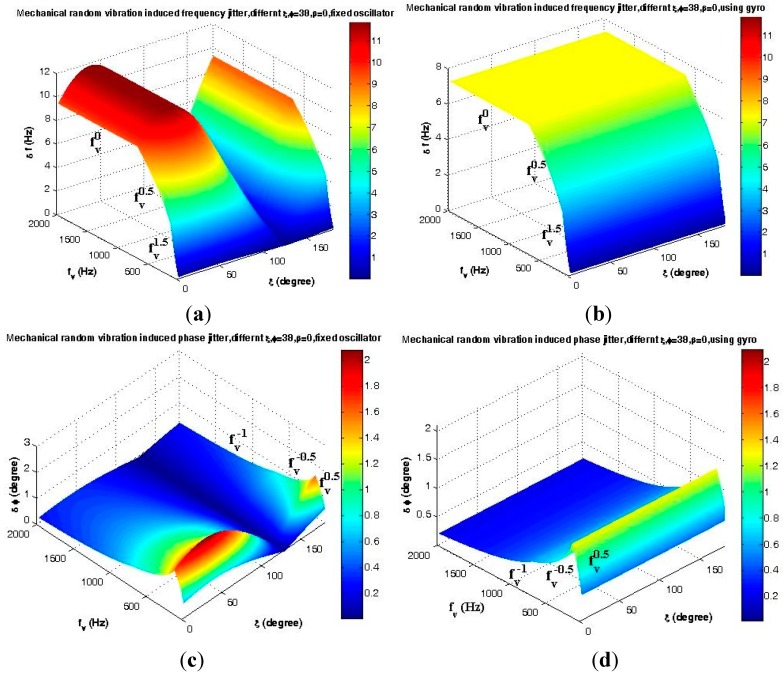
Analysis of the mechanical random vibration in the frequency domain for *β* = 0, |*φ*| = 38°. (**a**) Frequency jitter of a fixed oscillator; (**b**) Frequency jitter of the gyroscopic mounting; (**c**) Phase jitter of a fixed oscillator; (**d**) Phase jitter of the gyroscopic mounting.

Analyses of the mechanical random vibration-induced frequency and phase jitter in the frequency domain are shown in Figure 20 and Figure 21, before and after using the gyroscopic mounting*.*

These analyses have been performed with two approaches, first assuming that random vibration is in the same direction with the g-sensitivity vector, which means that the maximum disturbances are induced by the random vibration on the replica carrier, as shown in Figure 20. Since, in practice, random vibration can be applied in any direction, in the second approach, the elevation angle of this vector is assumed |*φ*| = 38° [2], and the effect of any possible random vibration, *i.e.*, 0 < *ξ* < 180°, has been analyzed, as shown in Figure 21. As can be seen in Figure 21a, the maximum drawback only occurs when β is sufficiently close to 90°.

##### Acoustical Random Vibration

According to Equations (32)–(34), the necessary parameters to calculate load *A* are the sound absorption coefficient *α* and Surface Area/Mass of crystal *k*; therefore, numerical analysis of acoustical random vibration depends on these mentioned parameters. Regardless of the random vibration of this load, it can be deduced that the response of the gyroscopic mounting to this load is the same as that to the mechanical random vibration analyzed previously.

Based on the dynamic loads on the trajectory in Section 5, the main signal loss occurs during atmospheric flight. In this part of the trajectory, the dominant disturbance source of FLL is acoustic load. In order to prevent signal loss during the ascent flight, it is necessary to reduce the influence of this load on the replica carrier in such a way that the total frequency deviation can be less than the 3*σ*_FLL_ threshold. This can be solved by using the gyroscopic mounting. This mounting has a significant effect on reducing the random vibration-induced disturbances regardless of the source that generated them.

## 7. Conclusions

The analysis of a highly dynamic GNSS receiver’s trajectory shows that the probability of signal loss is in the maximum state during atmospheric flight and in some special intervals of the upper atmospheric flight. The gyroscopic mounting method is analyzed on FLL as the dominant loop of highly dynamic receivers in order to prevent signal loss during the mission. The threshold of 1*σ*_FLL_ is 4.17 Hz. The analysis results show that during atmospheric flight, the maximum frequency deviation induced by steady-state load is 6.61 Hz for the crystal oscillator with parameters |*φ*| = 2° and *β* = 0. The gyroscopic mounting can be adopted to reduce this to 0.23 Hz. During the upper atmospheric flight, this jitter is 4.72 Hz, and the gyroscopic mounting reduces it to 0.16 Hz. In the case of sinusoidal vibration, during atmospheric flight and some special intervals in the upper atmospheric flight that maximum the jitter exits, the gyroscopic mounting reduces it from 2 Hz down to 1.57 Hz. In addition, the maximum gyroscopic mounting effect appears when *β* = 0, *ƒ* = 2 and *φ* = 2°. In this case, the frequency jitter is reduced from 1.32 Hz down to 0.070 Hz. The gyroscopic mounting affects the random vibration as the main disturbance source of FLL, regardless of the source, either mechanical or acoustical, during the atmospheric or upper atmospheric flight. The maximum effect appears when the load coincides with the g-sensitivity vector (*i.e.*, *ξ = φ*, *β* = 0, *φ* = 0), while the maximum drawback appears when the load is perpendicular to the g-sensitivity vector (*i.e.*, *ξ − φ* = 90° or *β* = 90°, |*φ*| = 38°). In the worst case, the FLL error is less than 3*σ*_FLL_ after using the gyroscopic mounting during 95% of the trajectory. In this way, the proposed mounting can effectively reduce the probability of signal loss, *i.e.*, the signal loss can be overcome in highly dynamic receivers by the aid of a gyroscopic mounting crystal oscillator. This method can provide theoretical support for new approaches related to highly dynamic GPS receivers.
